# Mechanistic Insights into Solvent-Mediated Halide-Specific Irreversible Transformation of Cu-MOF with Iodide Detection Capability

**DOI:** 10.1021/acs.inorgchem.4c04816

**Published:** 2025-02-13

**Authors:** Ahamad Irfan, Naga Venkateswara Rao Nulakani, Upendar Reddy Gandra, Robert Gyepes, Petr Henke, Martin Kubu, Jiří Mosinger, Youssef Belmabkhout, Ahsanulhaq Qurashi, Jiri Čejka, Russell Morris, Zhehao Huang, Mohamad Akbar Ali, M. Infas H. Mohideen

**Affiliations:** † Department of Chemistry, Khalifa University of Science and Technology, P.O. Box 127788, Abu Dhabi, United Arab Emirates; ‡ Center for Catalysis and Separations, Khalifa University of Science and Technology, P.O. Box 127788, Abu Dhabi, United Arab Emirates; § Department of Physical and Macromolecular Chemistry, Faculty of Science, Charles University, Hlavova 2030, 128 00 Prague 2, Czech Republic; ∥ Department of Materials and Environmental Chemistry, Stockholm University, SE-106 91 Stockholm, Sweden; ⊥ Department of Inorganic Chemistry, Faculty of Science, Charles University, Hlavova 2030, 128 00 Prague 2, Czech Republic; # Technology development Cell (TechCell), Technology Transfer Office (TTO), Mohammed VI Polytechnic University (UM6P), Ben Guerir 43150, Morocco; ∇ EaStCHEM School of Chemistry, University of St. Andrews, St. Andrews KY16 9ST, U.K.

## Abstract

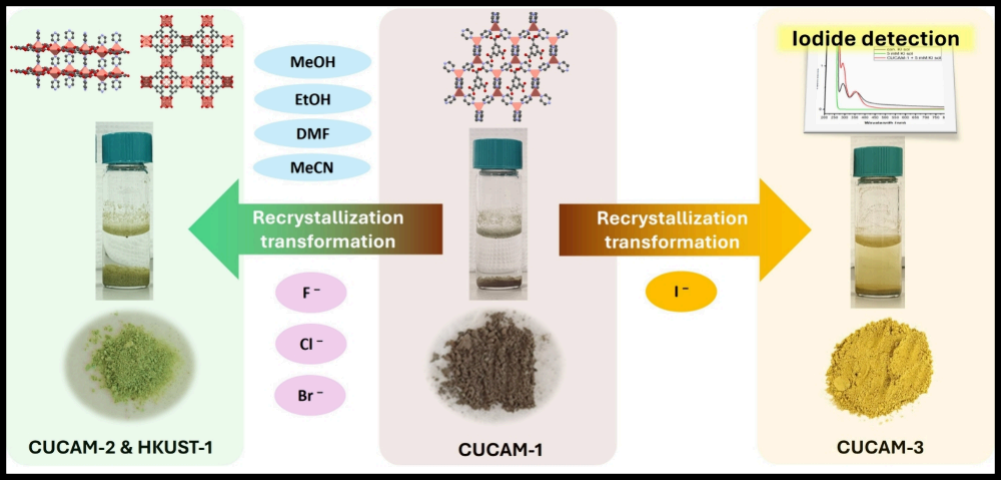

The fascinating feature of metal–organic frameworks is that they can respond to external stimuli, unlike other inorganic materials. This feature corresponds to the framework’s flexibility, which originates with the long-range crystalline order of the framework accompanied by cooperative structural transformability. We have synthesized a novel metal–organic framework comprised of Cu(I) nodes with pyrazine linkers and benzene-1,3,5-tricarboxylate acting as template anions, named CUCAM-1 [Cu(Py)_2_(BTC)]_n_. In the presence of polar solvent systems, CUCAM-1 undergoes an irreversible structural transformation to yield a mixed phase that consists of HKUST-1 [Cu_3_(BTC)_2_(H_2_O)_3_]_n_ and another CUCAM-2 [Cu(Py)(BTC)]_n_ MOFs, whose novel structure is successfully revealed by continuous rotation electron diffraction from the mixture. In this structural transformation, a new ligand exchange occurs where template anions become ligands, confirmed by single crystal X-ray analysis. Further, structural transformation and the mechanism are explained by ab initio molecular dynamics (AIMD) simulations. Interestingly, different halides (F^–^, Cl^–^, and Br^–^) can be accompanied to affect/control the composition of the second phase by favoring the formation of the HKUST-1 phase over CUCAM-2, which was evident by the powder X-ray diffraction studies. Furthermore, the structural transformation induced by I^–^ resulted in a colorimetric response due to the formation of a new MOF CUCAM-3, paving the way for use as an iodide detector.

## Abstract 2

Transforming MOF as iodide detector: A novel brown colored Cu(I) MOF, CUCAM-1 undergoes transformation into a green MOF mixture consisting of well-known HKUST-1 and new CUCAM-2 MOFs when exposed to polar solvents and halides except for iodide. When exposed to iodide, it transforms to yet another MOF, a yellow CUCAM-3, exhibiting its potential as iodide detector.

## 1 Introduction

Metal–organic frameworks (MOFs) have attracted increased interest due to their distinctive properties that make them potential candidates for versatile applications in a variety of fields.^1−5^ MOFs are an excellent platform for host–guest chemistry as they have shown great potential in applications like gas storage, separation, sensing, magnetism, and catalysis ion exchange.^6−12^ One of the unique features of MOFs is that they can respond to external stimuli such as pressure, temperature, molecules, ions, etc., which were not known in traditional inorganic porous materials.^13,14^ This makes the framework flexible and combines with long-range crystalline order to transform into cooperative structures, facilitating more room for discoveries.^15,16^ These flexible MOFs display attractive properties such as breathing phenomena and phase transitions that can exhibit reversible or irreversible structural transformability.^17,18^ Since many factors govern the framework’s flexibility, it is challenging to design the architecture or control the flexibility. This flexibility develops interesting structural responses toward external stimuli and improves their specific performance for the desired applications.

One aspect of flexibility in certain metal–organic frameworks (MOFs) is the ability to undergo cation and anion exchange.^19−21^ These processes can occur either through a single-crystal-to-single-crystal (SC-SC) transformation or recrystallization.^19^ The SC-SC transformation, typically considered a solid-state process, can be triggered by chemical and physical stimuli such as host–guest interaction, ligand exchange, temperature change, and light exposure.^22−24^ Conversely, the recrystallization process follows a solvent-mediated mechanism, involving the dissolution of the original MOF crystal and the precipitation of a new MOF crystal from the solution. The recrystallization process results in the restructuring of the initial crystal.^19^

Such transformations have been supportive of discovering and designing MOFs with sensing/detection capabilities. Several Co, Cu, Zn, Tb, and Eu MOFs have already been reported for applications such as halide sensing due to their distinctive host–guest chemistry and highly accessible voids/channels.^25−28^ In addition to traditional host–guest interaction-driven transformations, halide-triggered ligand-substitution-induced SC-SC transformations with a colorimetric response have also been observed and utilized for halide sensing applications.^22^ Despite these advances, practical limitations continue to create a significant demand for commercially viable rapid colorimetric sensor/detector material for halide detection.

It is well-known that halides, such as chloride, bromide, and iodide, are essential for various biological processes in the body. Among the halides, iodine is crucial for proper thyroid function and is commonly supplemented through iodized products like table salt.^22^ Radioactive waste also contains iodide, posing detection challenges.^29^ While mass spectrometry and atomic absorption spectroscopy can detect trace iodide levels, a rapid and practical iodide detector/sensor is needed, especially in underdeveloped regions.

Herein, we present a novel metal–organic framework, termed CUCAM-1, composed of Cu(I) nodes linked by pyrazine (Py) and benzene-1,3,5-tricarboxylate (BTC) template anions. This framework has been characterized by using various spectroscopic and diffraction techniques. CUCAM-1 undergoes an irreversible solvent-assisted structural transformation in the presence of polar solvent systems and certain halide anions (F^–^, Cl^–^, & Br^–^), resulting in a mixed phase comprising HKUST-1 and a new CUCAM-2 MOF. Interestingly, F^–^, Cl^–^, & Br^–^ could be accompanied to affect/control the relative composition of the second phase between CUCAM-2 and HKUST-1. The novel structure of the CUCAM-2 phase was successfully determined by using continuous rotation electron diffraction from the mixture. Additionally, the recrystallization process, which follows a solvent-mediated mechanism, was further elucidated through ab initio molecular dynamics (AIMD) simulations by using the CP2K program. Unlike other halides, the irreversible structural transformation induced by I^–^ resulted in a colorimetric response due to the formation of a new MOF, CUCAM-3. Inspired by previous research, this discovery encouraged further study and development of the novel CUCAM-1 as an iodide detection MOF that works by the less-explored method of solvent-assisted recrystallization.

## 2 Experimental Section

### 2.1 Synthesis of CUCAM-1 [Cu(Py)_2_(BTC)]_n_

A solution containing Cu(NO_3_)_2_·3H_2_O (241 mg, 1 mmol), pyrazine (Py) (160 mg, 2 mmol), benzene-1,3,5-tricarboxylic acid (H_3_BTC) (210 mg, 1 mmol), 7 mL of H_2_O, and 3 mL of MeOH was prepared in a Teflon-lined autoclave and heated to 180 °C for 24 h. Orange-brown crystals were harvested and air-dried (Yield: 85%). Elemental analysis for calculated formula [C = 43.3% (47.39%), H = 0.04% (2.57%), N = 11.3% (13.00%)].

### 2.2 Synthesis of the CUCAM-2 [Cu(Py)(BTC)]_n_ and HKUST-1 [Cu_3_(BTC)_2_(H_2_O)_3_]_n_ Mixture

An amount of ∼10 mg of CUCAM-1 was taken in a 7 mL scintillation vial along with 5 mL of a polar solvent (ethanol, methanol, acetonitrile, or dimethylformamide, etc.) or an aqueous solution of fluoride, chloride or bromide. The mixture was sonicated for 1 min and kept undisturbed which yielded a blue–green mixture of CUCAM-2 and HKUST-1 which was then obtained by vacuum filtration and air-dried.

### 2.3 Synthesis of CUCAM-3

An amount of ∼10 mg of CUCAM-1 was placed in a 7 mL scintillation vial along with 5 mL of aqueous iodide solution. The mixture was sonicated for 1 min and kept undisturbed which yielded yellow colored CUCAM-3 which was then obtained by vacuum filtration and air-dried.

### 2.4 Single-Crystal X-ray Diffraction

Single-crystal X-ray diffraction data were collected on a Bruker D8 VENTURE diffractometer using MoK_α_ primary radiation (λ = 0.71073 Å). The sample specimen was cooled to 150 K using an Oxford Cryostream Cooler. The collected data were processed by diffractometer software. The phase problem was solved by intrinsic phasing (SHELXT)^30^ and the structure model was refined by full-matrix least-squares on F^2^ (SHELX-2018/3).^31^ All non-hydrogen atoms were refined anisotropically; hydrogen atoms were placed into idealized positions and refined isotropically. Electron maxima appearing in voids due to solvent molecules could neither be described by any plausible chemical model nor could be refined satisfactorily, and hence their contribution was subtracted by the SQUEEZE procedure^32^ implemented in the PLATON program.^33^

### 2.5 Powder X-ray Diffraction

PXRD measurements were carried out at room temperature on a Bruker D8 ADVANCE diffractometer at 40 kV, 30 mA for Cu *K*α (λ = 1.5418 Å), with a step size of 0.05° in 2θ.

### 2.6 Elemental Analysis

Elemental analysis was performed with a Thermo Scientific Flash 2000 instrument.

### 2.7 Fourier Transform Infrared Spectroscopy

FT-IR spectra (4000–600 cm^–1^) were recorded on a Thermo Scientific Nicolet 6700 apparatus.

### 2.8 Transmission Electron Microscopy

Samples for transmission electron microscopy observations were dispersed in acetone. A droplet of the suspension was transferred to a carbon-coated copper grid. The observation was performed on a JEOL JEM2100 microscope and operated at 200 kV (Cs 1.0 mm, point resolution 0.23 nm). Images were recorded with a Gatan Orius 833 CCD camera (resolution 2048 × 2048 pixels, pixel size 7.4 μm) under low dose conditions.

### 2.9 Continuous Rotation Electron Diffraction

Electron diffraction patterns were recorded with a Timepix pixel detector QTPX-262k (512 × 512 pixels, pixel size 55 μm, Amsterdam Sci. Ins.). Continuous rotation electron diffraction (cRED) data were collected using the software *Instamatic*.^34^ A single-tilt tomography holder was used for the data collection, which could tilt from −70° to +70° in the TEM. The aperture used for cRED data collection was about 1.0 μm in diameter. The speed of the goniometer tilt was 0.45° s^–1^, and the exposure time was 0.5 s per frame. Data was collected within 5 min to minimize the beam damage and maximize the data quality. The data was processed using the *XDS* package.^35^ The framework structure determination and final refinement were done using the Shelx-2014.^36^

### 2.10 Gas Adsorption

Low-pressure gas sorption measurements were performed on a fully automated 3Flex high-resolution gas adsorption analyzer (Micromeritics) at relative pressures up to 1 atm. The cryogenic temperatures were controlled using liquid nitrogen and argon baths at 77 and 87 K, respectively.

### 2.11 UV–Visible Spectroscopy

UV–vis spectra measurements were performed on a PerkinElmer LAMBDA 1050+ UV/vis/NIR Spectrophotometer.

### 2.12 Thermogravimetric Analysis

Thermogravimetric analysis measurements were performed on a PerkinElmer STA 6000 thermal analyzer, under a nitrogen atmosphere (flow = 20.0 cm^3^ min^–1^, heating rate = 5 °C min^–1^)

### 2.13 Computational Details

The Born–Oppenheimer molecular dynamics (BOMD) simulations, based on density functional theory (DFT), were conducted using the CP2K 6.1.0 software package.^34,35^ For this study, the Perdew–Burke–Ernzerhof (PBE) functional was employed, incorporating Grimme’s DFT-D3 semiempirical dispersion correction to accurately capture weak interactions.^36,37^ The electronic structure was treated using a double-ζ valence polarization (DZVP-MOLOP)^38^ Gaussian basis set for the valence electrons, while Goedecker–Teter–Hutter (GTH) norm-conserving pseudopotentials were applied to represent the core electrons.^39,40^ An energy cutoff of 280 Ry was used for the plane wave basis set and 40 Ry for the Gaussian basis set. The BOMD simulations were carried out within an NVT ensemble at constant volume and a temperature of 300 K, utilizing a time step of 1.0 fs. Periodic supercells with side lengths of 25 Å were employed to model the gas-phase reaction.

## 3 Results and Discussion

### 3.1 Topological Analysis

**Figure 1:**
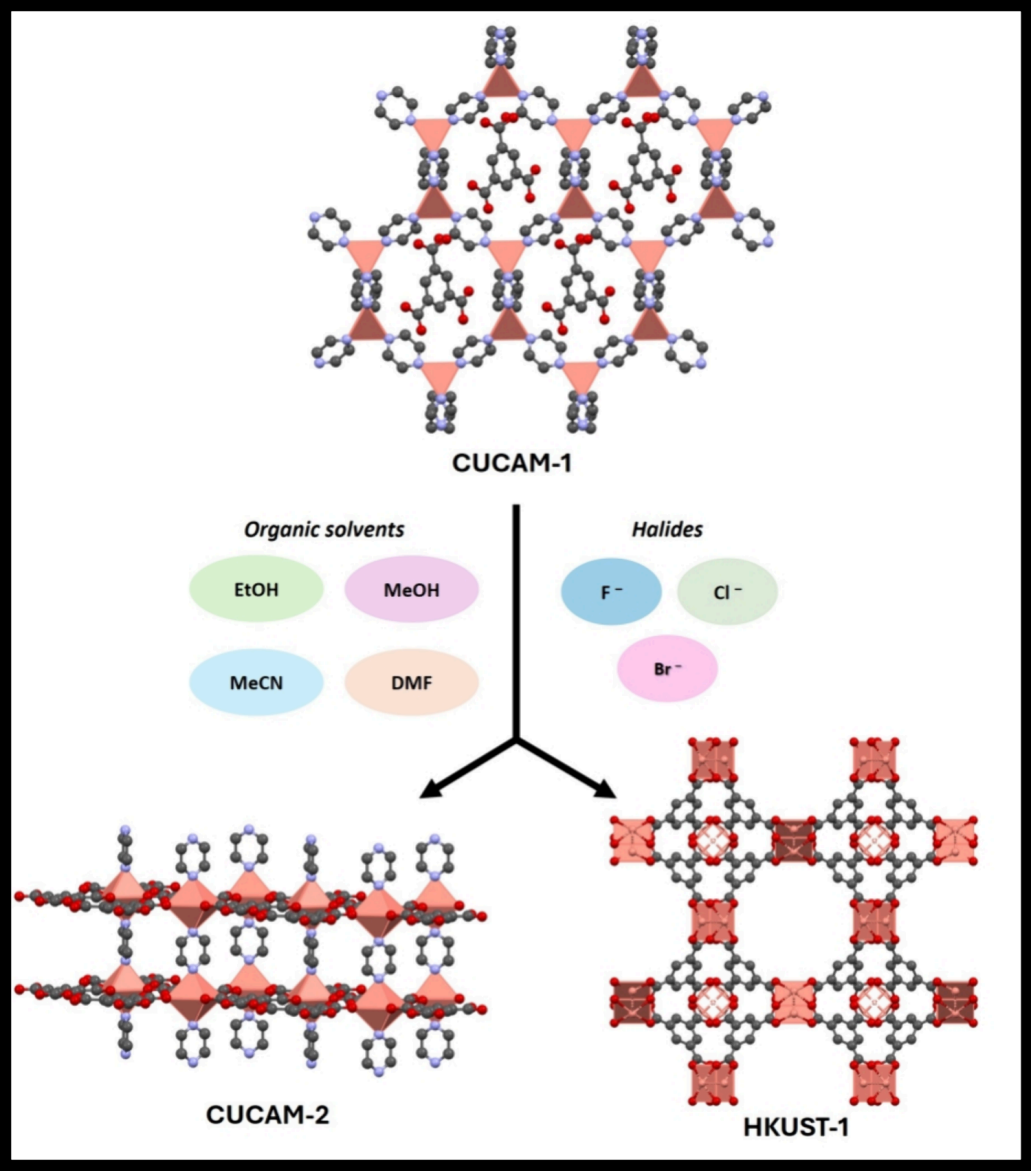
Structural transformation of CUCAM-1 to CUCAM-2 and HKUST-1.

The single crystal X-ray diffraction studies of CUCAM-1 [Cu(Py)_2_(BTC)]_n_ revealed that the framework adopts the triclinic crystal system and comprises Cu(I) ions as metal nodes and pyrazine molecules as ligands where Cu ions adapt to tetrahedral geometry with an oxidation number of +1. The assembly of four connected Cu nodes with pyrazine (two-connected linker) molecules resulted in the 3D framework with 2D channels with a diamondoid topology (Figures 1 and S1). Although H_3_BTC in the form of BTC is usually expected as a ligand, in this case, it has the role of a template that triggers the formation of bigger channels within the framework. One of the carboxylates of H_3_BTC has deprotonated (H_2_BTC) to compensate for the framework’s charge balance to maintain the MOF’s structural neutrality. Hence, H_2_BTC acts as a template and a charger compensator, further supported by previous studies.^41^ Further, the phase purity of the MOF was confirmed by Powder X-ray diffraction (PXRD) and elemental analysis (Figure S2).

In the presence of different solvent systems CUCAM-1 undergoes a color change accompanied by a phase transition, which has been identified by X-ray powder diffraction (Figures 1 and S3). The Cu(I) in the initial brown solid oxidizes to Cu(II) by becoming bluish-green in certain organic solvent systems such as ethanol, methanol, acetonitrile, dimethylformamide, etc., caused by an irreversible solvent-mediated recrystallization process.^19^ Interestingly, the framework integrity remains unchanged in other solvent systems such as water, chloroform, toluene, dichloromethane, etc. (Figure S4).

**Figure 2:**
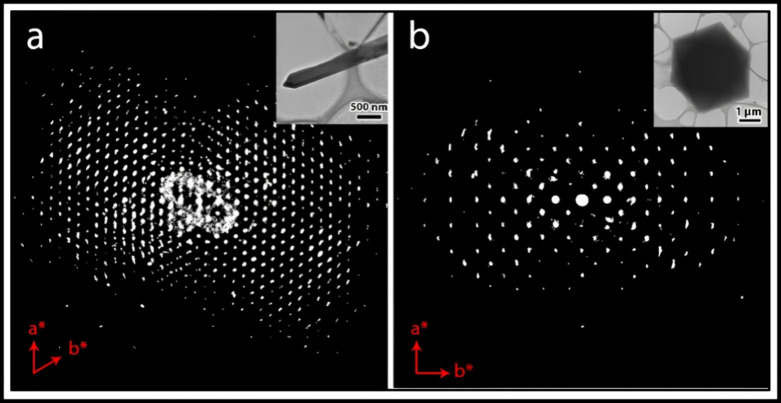
3D Reconstructed reciprocal lattice of (a) CUCAM-2, and (b) HKUST-1. Inset is the crystal from which the cRED data were collected.

The comparison of the infrared spectra of the brown solid with the green solid shows a considerable change in the carboxylic peaks (Figures S5 and S6). The peak at 1713 cm^–1^, which corresponds to the stretching vibrations of the free carboxylates of H_3_BTC, shifted to 1689 cm^–1^, suggesting possible interactions between carboxylates and metal ions. Literature analysis suggests that the carboxylate stretching peak of the −COO^–^ bonded to Cu ions lies around 1690 cm^–1^.^16^ By taking advantage of the strong interaction between electrons and matter, we applied continuous rotation electron diffraction (cRED)^16,39−41^ for single-crystal analysis of the tiny green crystals. It revealed that two phases coexist in the bluish-green compound after the structural transformation of CUCAM-1. One phase comprises nanorod crystals with a diameter of 250–400 nm, and a length of 5–10 μm, which has a hexagonal unit cell with the parameters of *a* = 16.35 Å, and *c* = 6.93 Å. Meanwhile, the other contains octahedral nanocrystals with a diameter of 1–5 μm, with a face-centered cubic unit cell and *a*-parameter = 27.11 Å (Figure 2). Each of the single crystals was analyzed, and both structural models were determined ab initio, with further refinement against cRED data (Figures S7 and S8, and Table S2; see Supporting Information for more details). As a result, the nanorod crystals were identified to be a unique new MOF and were named CUCAM-2 [Cu(Py)(BTC)]_n_, and the octahedral nanocrystal was found to be HKUST-1 [Cu_3_(BTC)_2_(H_2_O)_3_]_n_. The difference between these two phases, simply stated, is that CUCAM-2 has the pillaring pyrazine molecules between the layers, whereas HKUST-1 is devoid of this. Knowing the structures and the coexistence of two phases, the peaks in the PXRD pattern can thus be successfully assigned to both phases (Figure S9). This work further demonstrates the importance of electron diffraction for developing and discovering novel MOFs.^42^

CUCAM-2 is a mixed-linker MOF consisting of linking Cu(II) cations with BTC and pyrazines (Figure S10). It uniquely forms a 3D framework by pillaring 2D layers, where each BTC connects to three Cu(II) cations in the layers. Each pyrazine molecule connects two Cu(II) cations perpendicular to the layers and acts as a pillar to form a 3D framework (Figure S10d). Cu(II) cations adopt a distorted octahedral geometry that coordinates three trimesic acids and two pyrazines. Furthermore, the interlayer distance of CUCAM-2 is ca. 3.5 Å. This indicates that the structure is connected by labile coordination bonds and stabilized by π–π interactions, which could provide extra chemical stability under harsh conditions. In addition, the BTC molecules are partly connected through hydrogen bonding (Figure S10c, indicated by dash lines), similar to that observed for previously reported Ni-MOFs.^43,44^ Although ligand exchange is common in MOF chemistry, a template ending up as a ligand is unusual, and CUCAM-1 provides an example of a new type of ligand exchange. The major driving force for the structural transformation process here can be attributed to the changes in polarity brought about by the different solvents. The change in polarity can increase the nucleophilicity of the BTC anions which drives their nucleophilic attack toward Cu(I) ions and the eventual formation of CUCAM-2 with oxidized Cu(II) nodes. Specific polarity conditions can break the precipitation-dissolution equilibrium of CUCAM-1 which leads to the dissolution of CUCAM-1 and the formation of new CUCAM-2 and HKUST-1 crystals. This new growth continues until a new equilibrium is reached.^19^

### 3.2 AIMD Studies

**Figure 3:**
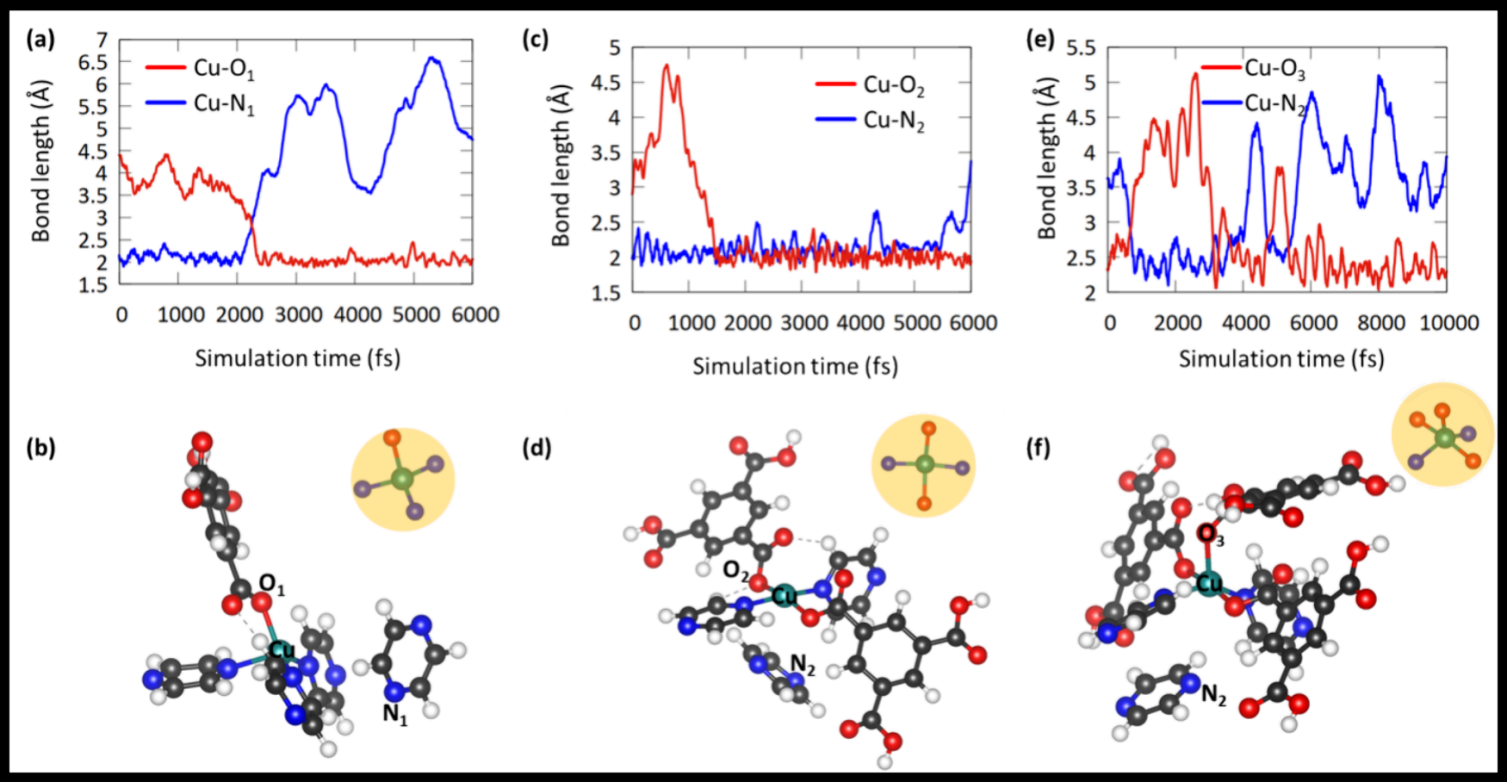
Variation of Cu–O and Cu–N bond lengths during the AIMD simulation and the final geometries of (a,b) Cu(Py)_3_(H_2_BTC), (c,d) Cu(Py)_2_(H_2_BTC)_2_, and (e,f) Cu(Py)_2_(H_2_BTC)_3_.

To gain deeper insights into the solvent-mediated transformation from CUCAM-1 to CUCAM-2 or HKUST-1, ab initio molecular dynamics (AIMD) simulations were carried out using the CP2K program.^34,45^ Further computational details are provided in the Supporting Information, Section 8. In a series of AIMD simulations, the Cu(Py)_4_ moiety sequentially interacted with H_2_BTC anions. Our computational results indicate that the H_2_BTC anion attacks the Cu(Py)_4_ moiety similar to the SN2 mechanism. Here, the H_2_BTC anion functions as a nucleophile, targeting the Cu metal center, while the opposing pyrazine molecule acts as the leaving group. The variation in bond length between the oxygen (O_1_) atom of the H_2_BTC anion, the nitrogen (N_1_) atom of pyrazine, and the Cu metal center is calculated and depicted in Figure 3a. It clearly shows the formation of a new bond (Cu–O_1_) between the O_1_ atom of the H_2_BTC anion and the Cu metal center, along with the breaking of the Cu–N_1_ bond after the 2250 fs time scale. Furthermore, it illustrates that the Cu–O_1_ bond stabilizes, exhibiting a bond length of around 2 Å, while the Cu–N_1_ bond length exceeds 4 Å as the pyrazine molecule moves away from the Cu metal center. Additionally, there is no evidence of the reformation of the Cu–N1 bond with the pyrazine ligand. A snapshot taken at the end of the AIMD simulation further supports the formation of the new Cu–O_1_ bond with that of the H_2_BTC anion and the breaking of the Cu–N_1_ bond, as shown in Figure 3b. The coordinating environment of the Cu with three pyrazine and one H_2_BTC ligand is visible in the inset of Figure 3b. It reveals that the equilibrium geometry of the resulting Cu(Py)_3_(H_2_BTC) anion exhibits a tetrahedral structure.

Subsequently, the tetrahedral Cu(Py)_3_(H_2_BTC) moiety sequentially interacts with the second and third H_2_BTC anions. The second H_2_BTC anion attacks the Cu metal center similarly to the first, replacing another pyrazine ligand and forming Cu(Py)_2_(H_2_BTC)_2_. The formation of the new Cu–O_2_ bond with the second H_2_BTC anion and the breaking of the Cu–N_2_(pyrazine) bond by the end of the AIMD simulation can be observed in Figure 3c. The central Cu atom displays square-planar-type bonding with two pyrazine and two H_2_BTC ligands, as shown in Figure 3d. At this stage, the model depicted in Figure 3d was taken as the initial geometry to explore the potential for rebonding of the leaving pyrazine with the Cu-metal center during the interaction of the third H_2_BTC anion with Cu(Py)_2_(H_2_BTC)_2_.

Figure 3e clearly demonstrates that the leaving pyrazine ligand from the previous step begins to interact with Cu during the AIMD simulation with the third H_2_BTC. The Cu–N_2_ bond forms after 800 fs and remains intact until 5000 fs. However, the third H_2_BTC anion begins interacting with the Cu metal center, and a new Cu–O (Cu–O_3_) bond forms at 3000 fs. The Cu–O_3_ bond stabilizes after 5000 fs, while the Cu–N_2_ bond breaks, and the corresponding pyrazine ligand moves away from the central Cu atom. Additionally, the third H_2_BTC anion does not replace any of the other pyrazine ligands at the Cu metal center, forming Cu(Py)_2_(H_2_BTC)_3_ with a square pyramidal-like structure. In the Cu(Py)_2_(H_2_BTC)_3_ structure, two of the H_2_BTC anions and two pyrazine ligands occupy opposite corners of the base of the square, while one of the H_2_BTC anions is positioned above the basal plane of the square.

**Figure 4:**
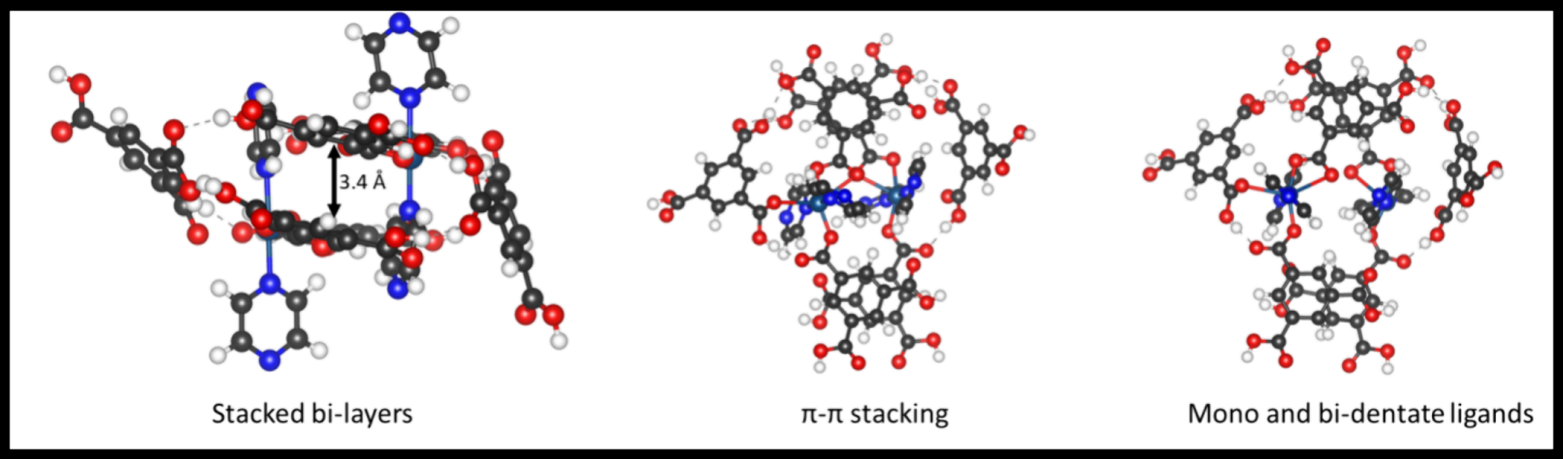
Comparison of the structural features between the computed and experimental models of CUCAM-2.

Finally, the interaction of two such Cu(Py)_2_(H_2_BTC)_3_ moieties results in the formation of a complex with structural features similar to those of CUCAM-2, as presented in Figure 4. Specifically, two Cu(Py)_2_(H_2_BTC)_3_ moieties stack together through π–π interactions between the benzene rings of the BTC units. The computed interlayer distance is approximately 3.4 Å. In the final complex, one of the carboxylic groups of the BTC ligands acts as a bidentate ligand, while the other two serve as monodentate ligands, as shown in Figure 4. Furthermore, the CUCAM-2 exhibits structural characteristics similar to H1 and H2 of a previous study.^46^ Ramirez and co-workers experimentally proved that the layered structures (H1 and H2) directly convert to HKUST-1 upon ethanol treatment. It indicates that the CUCAM-2 may follow a similar transformation pathway. Collectively, these results clearly corroborate our experimental findings, confirming the solvent-mediated recrystallization transformation of CUCAM-1 to CUCAM-2 and HKUST-1.

### 3.3 Anion Exchange-Assisted Recrystallization Transformation and Selective Iodide Detection

Initial adsorption studies showed that the fully evacuated as-synthesized CUCAM-1 (brown solid) did not adsorb N_2_ and Ar at 77 and 87 K, respectively. This shows that channels are blocked by the template anions, which was anticipated from the analysis of the crystallographic structure. Therefore, attempts were made to exchange these template anions with different types of halides (e.g., F^–^, Cl^–^, Br^–^, & I^–^) to probe for adsorption. It was observed that these halides induced a phase transition. When CUCAM-1 was soaked in an aqueous solution of alkali metal halides of concentration as low as 5 mM, F^–^, Cl^–^, and Br^–^ induced the structural transformation to the green solid, which is a mixture of HKUST-1 & CUCAM-2 as observed when immersed in polar solvents such as MeOH, EtOH, DMF and MeCN. The rapidness of the change followed the order F-, Cl-, and Br- which could be simply attributed to the relative solubilities of these anion salts and, therefore, their availability in the solution (Figure S12).

**Figure 5:**
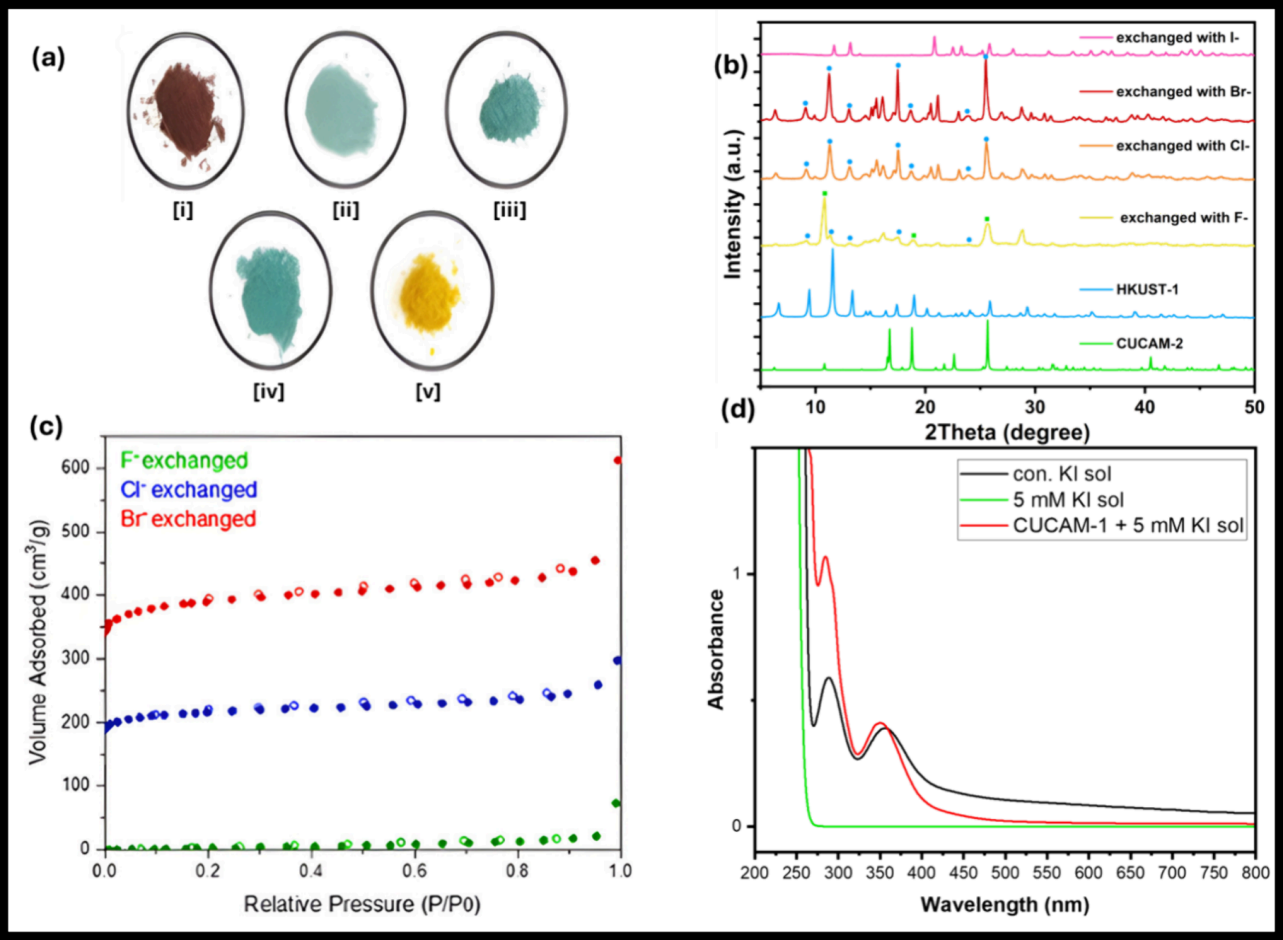
(a) Appearance of [i] CUCAM-1 only and CUCAM-1 in the presence of [ii] Fluoride [iii] Chloride [iv] Bromide [v] Iodide. (b) PXRD patterns of CUCAM-1 after exchanging with different halides in comparison to pattern of pure CUCAM-2 and HKUST-1 indicating their varying presence in the exchanged samples and the distinct CUCAM-3 phase obtained after I^–^ exchange (c) N_2_ adsorption isotherms on Green solid at 77 K after exchanged with F^–^, Br^–^, & I^–^ respectively and evacuation at 398 K. (d) UV–vis spectra of CUCAM-1 in 5 mM KI solution showing triiodide ion peaks around 285 and 350 nm, similar to those in a concentrated KI solution, while a 5 mM KI solution alone shows no peaks.

Unlike F^–^, Cl^–^, and Br^–^, iodide (I^–^) triggers a different irreversible phase transition, resulting in the formation of a yellow solid (CUCAM-3), as confirmed by PXRD (Figure 5a,b). This transformation’s speed and the yellow color’s intensity increase with higher iodide concentrations. As iodide ion is known to induce a redox-associated transformation,^22^ the plausible reaction here involves Cu(I) initially oxidizing to Cu(II), which then oxidizes I^–^ in the solution to I_2_. The I_2_ subsequently reacts with the remaining I^–^ to form I_3_^–^, resulting in the characteristic orange-yellow color of the iodine solution.

After template anions were exchanged with different halides, the resulting compounds (green solids, HKUST-1 + CUCAM-2, and yellow solid, CUCAM-3) were subjected to adsorption studies. The permanent porosity of the green solid was confirmed by N_2_ adsorption, giving rise to different surface areas and pore volumes with varying anions that have been used to exchange. According to the porosity measurements, the Br- exchanged sample has the highest surface area and pore volume (1540 m^2^/g, 0,7 cc/g) which is almost double the surface area and pore volume which was recorded for Cl^–^ exchanged sample (854 m^2^/g, 0.4 cc/g). F^–^ exchanged sample had a very small uptake of N_2_ (Figure 5c and Table S3). This behavior can be attributed to the amount of HKUST-1 formed during the structural transformation over the amount of CUCAM-2 formed. As we know, HKUST-1 is a highly porous material, whereas CUCAM-2 was anticipated to be nonporous as per the crystallographic data. Therefore, the high uptake of N_2_ should be governed by the amount of HKUST-1 formed during the phase transition. Although the mechanism is not clear, from the adsorption results, it can be concluded that the Br^–^ exchanged sample has a higher tendency to rearrange into HKUST-1 than CUCAM-2, whereas the Cl^–^ exchanged sample has formed a reasonable amount of both materials and F^–^ exchanged samples must have favored the formation of CUCAM-2 over HKUST-1. As evident by the adsorption studies, it is clear that halides control the composition of phase-2 by favoring the formation of one MOF over the other, and these results were repeatedly confirmed. These observations were also supported by the difference in the relative presence of these phases in the PXRD pattern of the exchanged samples. As shown in Figure 5b, the major peaks associated with the HKUST-1 phase (blue dots) exhibit an intensity increase on moving from F^–^ to Br^–^ exchanged samples. Additionally, the CUCAM-2 specific peaks (green squares) mainly visible in the F^–^ exchanged sample either diminish or show a shift to the HKUST-1 peak positions in the Cl^–^ and Br^–^ exchanged samples. Hence, the PXRD patterns further support the higher tendency of the latter exchanged sample to rearrange to HKUST-1 compared to the predominantly CUCAM-2 present F^–^ exchanged sample.

In addition to the N_2_ uptake study, the thermal stability of the samples was analyzed by thermogravimetric analysis (Figure S11). CUCAM-1 was found to be stable up to 523.15 K while the CUCAM-2 & HKUST-1 mixture and CUCAM-3 had lower stabilities with degradation onset around 498.15 K. However, the residual weight of the CUCAM-2 & HKUST-1 mixture was higher (∼85%) than that of both CUCAM-1 (∼50%) and CUCAM-3 (∼48%).

The crystallographic structure of CUCAM-3 could not be solved, as the quality of the crystals after I^–^ exchange was low. Moreover, after full evacuation, CUCAM-3 did not adsorb N_2_ and Ar at 77 and 87 K, respectively, demonstrating its nonporosity. As CUCAM-1 undergoes an irreversible phase change to the bright yellow structure of CUCAM-3, it demonstrates potential as an iodide detection material. While an aqueous KI solution is colorless due to its lack of specific absorbance in the visible range, CUCAM-1 enables colorimetric iodide detection by amplifying its presence through triiodide formation. Even though KI solution can exhibit triiodide-specific absorbance peaks around 288 and 356 nm at very high concentrations due to oxidation, this is not usually observed at low concentrations, such as 5 mM. However, with the addition of just 10 mg of CUCAM-1, even at low iodide concentrations, the solution turns yellow due to the liberation of triodide (I_3_^–^) as the MOF undergoes a phase change to yellow CUCAM-3. This visible color change is supported by the UV–vis spectra of the CUCAM-1 exposed KI solution, which shows enhanced triiodide ion absorption peaks at the wavelengths mentioned (Figure 5d). The limit of detection (LOD) calculation was done using the formula (3σ/slope) which required the slope of the calibration plot (Figure S14) and the standard deviation (σ) of blank readings. A LOD of 50.2 μM was determined for iodide detection by CUCAM-1. A comparison of this LOD value to other MOF-based colorimetric iodide sensors is provided in Table S4. The solid-state UV–vis spectra of the MOF before and after immersion in KI solution also reflect this transformation, with a significant drop in absorption beyond 500 nm (Figure S13), indicating the change from brown to yellow. This amplifying effect of CUCAM-1 allows for the detection of iodide at very low concentrations.

## 4 Conclusions

We successfully synthesized a novel diamondoid MOF (CUCAM-1), featuring Cu(I) nodes with pyrazine linkers and H_2_BTC acting as a template. Upon exposure to certain organic solvents such as dimethylformamide, ethanol, methanol, and acetonitrile, CUCAM-1 undergoes an irreversible structural transformation due to the oxidation of Cu(I) to Cu(II), resulting in a mixed phase composed of HKUST-1 and CUCAM-2. Notably, the template anion, H_2_BTC, exhibits a high affinity for Cu nodes, transitioning from a template to a linker (BTC) in newly formed CUCAM-2 and HKUST-1. This discovery reveals a novel type of ligand exchange where the template becomes a ligand. Furthermore, halides (F^–^, Cl^–^, and Br^–^) act as chemical triggers that influence the formation of HKUST-1 and CUCAM-2, as demonstrated by adsorption studies. Halide choice controls the composition of the mixed phase and the formation of HKUST-1 to CUCAM-2. In contrast to other halides, CUCAM-1 exhibits a colorimetric response upon adding iodide (I^–^), leading to the formation of a third phase, CUCAM-3, rendering CUCAM-1 as a potential iodide detection material. Ab Initio Molecular Dynamics (AIMD) simulation studies were conducted to validate the experimental findings of solvent- and anion-mediated irreversible transformation of CUCAM-1 to CUCAM-2 and HKUST-1. This study highlights the potential of exploring novel MOF transformations through template and ligand exchanges, particularly where template molecules transition into active ligands.

## Data Availability

Data are available within the article or in the ESI file.

## Supporting Information Available

Single crystal X-ray data and structure figures, additional powder X-ray diffraction patterns, infrared spectra, continuous rotation electron diffraction data and figures, surface area and pore volume data, thermogravimetric analysis plots, MOF transformation figures, UV–visible spectra, absorbance vs concentration calibration curve, and LOD comparison table (PDF)
