# Supplementary material for: Mechanistic Insights into Solvent-Mediated Halide-Specific Irreversible Transformation of Cu-MOF with Iodide Detection Capability
Source: Inorg Chem. 2025 Feb 13;64(7):3326–34. doi: 10.1021/acs.inorgchem.4c04816 (PMC11863367; doi:10.1021/acs.inorgchem.4c04816)
Supplement: Supplementary file 1 — ic4c04816_si_001.pdf [file ic4c04816_si_001.pdf]

Mechanistic Insights into Solvent-Mediated Halide-Specific Irreversible Transformation of Cu-MOF with Iodide Detection capability

Ahamad Irfan[a,b], Naga Venkateswara Rao Nulakani[a], Upendar Reddy Gandra[a], Robert Gyepes[e], Petr Henke[e], Martin Kubu[c], Jiří Mosinger[e], Youssef Belmabkhout[f], Ahsanulhaq Qurashi[a,b], Jiri Čejka[c], Russell Morris[c,g] Zhehao Huang[d]*, Mohamad Akbar Ali [a,b]*, M. Infas H. Mohideen[a,b,c]*

[a]*Department of Chemistry, Khalifa University of Science and Technology, Abu Dhabi, P.O. Box 127788, United Arab Emirates. E-mail: mohamed.mohideen@ku.ac.ae; akbar.mohamad@ku.ac.ae*

[b]*Center for Catalysis and Separations, Khalifa University of Science and Technology, Abu Dhabi P.O. Box 127788, United Arab Emirates.*

[c]*Department of Physical and Macromolecular Chemistry, Faculty of Science, Charles University in Prague, Hlavova 2030, Prague 2, 128 00, Czech Republic.*

[d]*Department of Materials and Environmental Chemistry, Stockholm University, SE-106 91 Stockholm, Sweden. E mail: zhehao.huang@mmk.su.se*

[e]*Department of Inorganic Chemistry, Faculty of Science, Charles University in Prague, Hlavova 2030, Prague 2, 128 00, Czech Republic.*

[f]*Technology development Cell (TechCell), Technology Transfer Office (TTO), Mohammed VI Polytechnic University (UM6P), Ben Guerir 43150, Morocco.*

[g]*EaStCHEM School of Chemistry, University of St. Andrews, St. Andrews KY16 9ST, Scotland.*

Table of Contents

1. Single Crystal X-ray Data and Structure Figures 3
2. Powder X-ray Diffraction 4
3. Infrared analysis 6
4. Continuous Rotation Electron Diffraction 7
5. Low-Pressure Gas Adsorption Measurements 11
6. Thermogravimetric analysis 12
7. Anion Exchange-Assisted Recrystallization Transformation & Selective Iodide Detection 13
8. Reference 14

1. Single Crystal X-ray Data and Structure Figures

Table S1: Crystal data and structure refinement for CUCAM-1

Identification code	**mihmmof_stam1_t40_sq**
Empirical formula	$C_{17}H_{11}CuN_4O_6$
Formula weight	430.84
Crystal system, space group	Triclinic, *P*-1
Unit cell dimensions	a = 10.3037(9) A alpha = 112.792(5) °<br>b = 10.5888(10) A beta = 107.908(5) °<br>c = 10.6888(11) A gamma = 101.682(5) °
Volume	951.82(16) Å^3
Z, calculated density	2, 1.643 Mg m^{-3}
F(000)	436
Temperature (K)	150.0(2)
Radiation type	Mo*K*α
Absorption coefficient	1.205 mm^{-1}
Absorption correction	Multi-scan
Max and min transmission	0.110 and 0.024
Crystal size	0.253 x 0.205 x 0.177 mm
Shape, color	Plate, translucent orange
θ range for data collection	2.233 to 27.549 deg.°
Limiting indices	$-13 \le h \le 13$, $-13 \le k \le 13$, $-13 \le l \le 13$
Reflection collected / unique / observed with $I > 2\sigma(I)$	31222 / 4365 [R(int) = 0.0691]
Completeness to θ_{max} = 68.5°	99.9 %
Refinement method	Full-matrix least-squares on F^2
Data / restraints / parameters	4365 / 0 / 264
Final *R* indices [$I > 2\sigma(I)$]	*R1 = 0.0612, wR2 = 0.1599*
Final *R* indices (all data)	*R1 = 0.0722, wR2 = 0.1601*
Weighting scheme	$[\sigma^2(F_o^2) + (0.1568P)^2 + 9.9294P]^{-1*}$
Goodness-of-fit	1.152
Largest diff. peak and hole	2.549 and -0.544 e Å^{-3}

$^*P = (F_o^2 + 2F_c^2)/3$

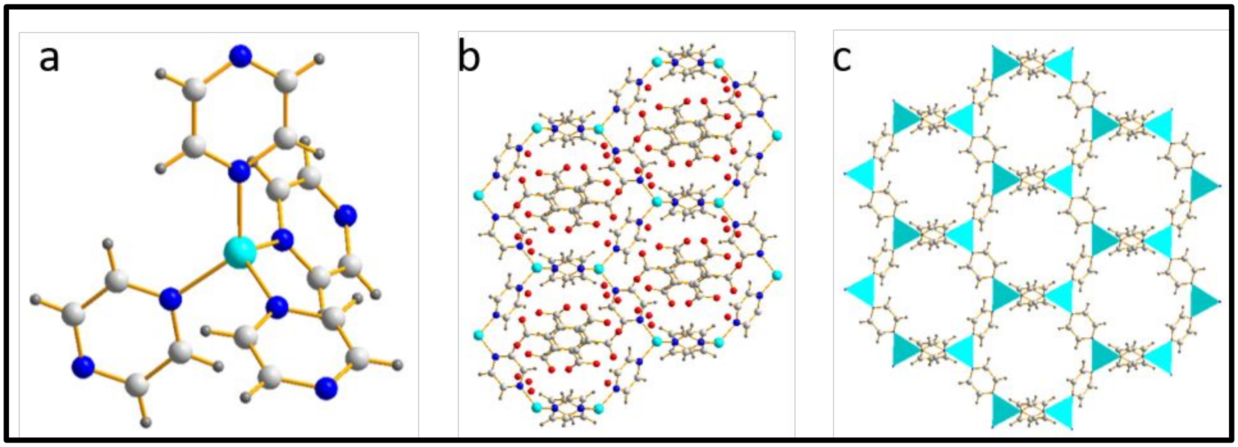

Figure S1: Structure of CUCAM-1; (a) Tetrahedral coordination of the metal node (b) Channels of the framework including its template anions (c) Channels of the framework by removing the template anions for clarity.

2. Powder X-ray Diffraction

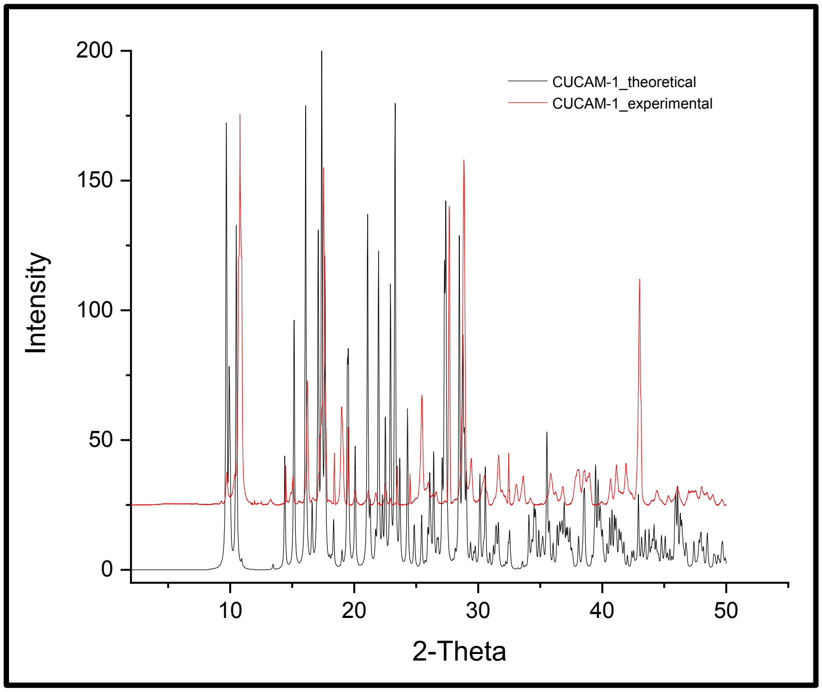

Figure S2. The comparison between experimental and calculated powder X-ray diffraction (PXRD) patterns for CUCAM-1, confirms the phase purity of the as-synthesized sample.

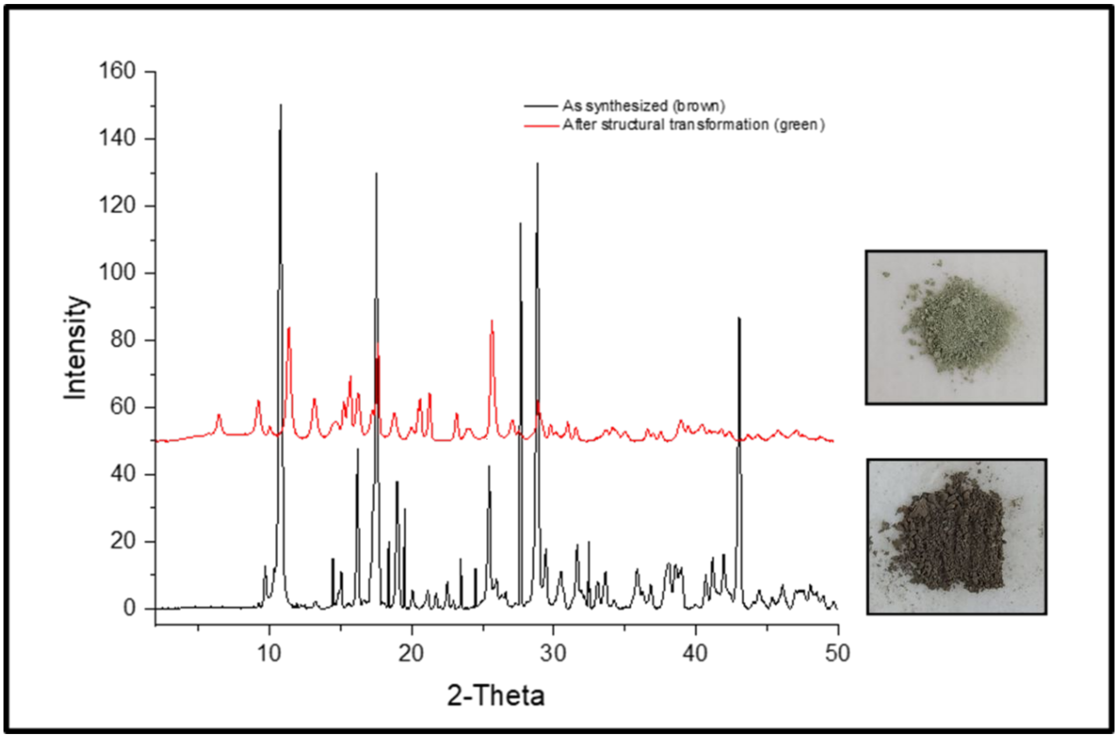

Figure S3. Experimental powder X-ray diffraction pattern of as-synthesized material-CUCAM-1 (brown solid) and material after the structural transformation (green solid).

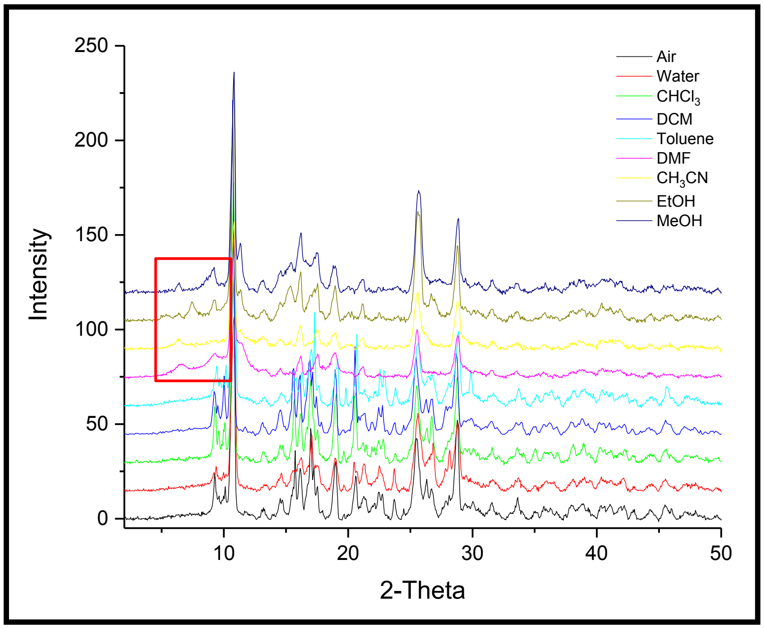

Figure S4. PXRD patterns of CUCAM-1 after soaking in different solvents for 24 h, indicating phase transition in some solvents.

3. Infrared analysis

IR measurements performed on the CUCAM-1 $[Cu(Py)_2(BTC)]_n$ sample clearly shows the presence of a free carboxylate group of the Benzene-1,3,5-tricarboxylic acid, with the characteristic νC=O band (1713 cm-1), and this peak has been shifted to 1689 cm^{-1} in MOF-b sample.

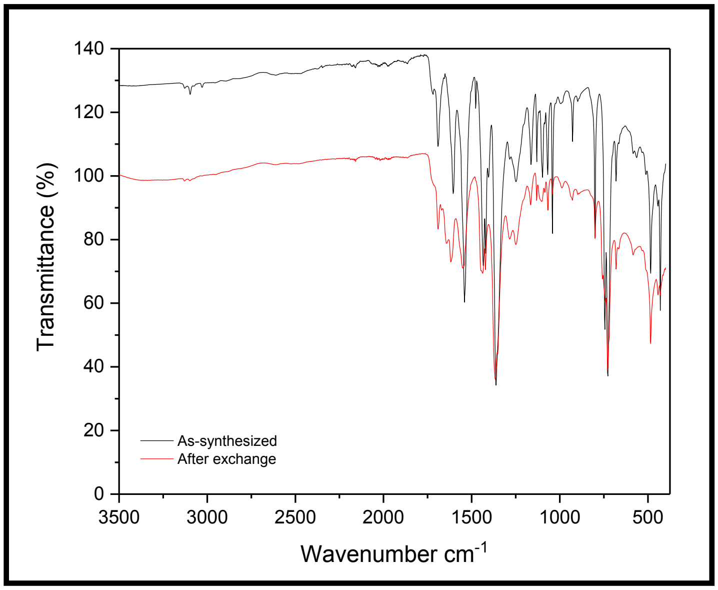

Figure S5. Comparison of IR spectra of the as-synthesized-brown solid and after solvent exchanged-green solid

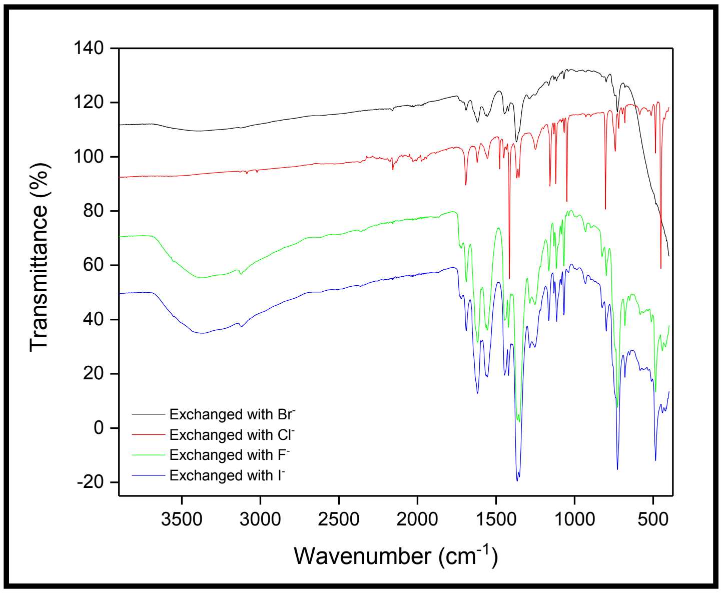

Figure S6. Comparison of IR spectra of CUCAM-1 sample after exchanged with halides.

4. Continuous Rotation Electron Diffraction

The 3D reconstructed reciprocal lattice from the cRED data shows CUCAM-2 $[Cu(Py)(BTC)]_n$ has a hexagonal unit cell with the parameters of a = 16.35 Å, and c = 6.93 Å. From 2D slice cuts of the 3D reciprocal lattice at *hhl* and *h-hl* planes (Figures S7), the reflection condition can be deduced as 00*l*: *l* = 2*n*. Thus, there are several possible space groups for CUCAM-2: $P6_3$ (No. 173), $P6_3/m$ (No. 176), and $P6_322$ (No. 182). The cRED dataset of CUCAM-2 has a resolution of 0.70 Å for structure solution. The data was processed by using *XDS* package.[1] The completeness is 99.6% and the R_{int} value is 0.270. The framework structure of CUCAM-2 was determined by direct methods using the program Shelx-2014[2] in space group $P6_3/m$. All the Cu, C, N, and O atoms were found directly. The final refinement was done by using Shelxl-2014, and data converged to R_1 = 0.217. From the cRED data of octahedra nanocrystals, we found it has a face centered cubic unit cell with the parameters of a = 27.11 Å. The reflection conditions can be deduced from the 3D reciprocal lattice (Figure S8) as *hkl*: $h + k = 2n$, $h+l = 2n$, $k+l = 2n$; 0*kl*: $k = 2n$, $l = 2n$; *hhl*: $h + l = 2n$; 00*l*: $l = 2n$. The possible space groups are *F*23 (No. 196), *Fm*-3 (No. 202), *F*432 (No. 209), *F*-43*m* (No. 216), and *Fm*-3*m* (No. 225). The space group *Fm*-3*m* with the highest symmetry was chosen for further structure determination. With a resolution of 1.30 Å, a completeness of 95.8%, and a R_{int} of 0.271, the structure of the octahedra nanocrystals was *ab initio* determined. The final refinement converged to R_1 = 0.173. The structural model shows that the octahedra nanocrystals have the HKUST-1 $[Cu_3(BTC)_2(H_2O)_3]_n$ structure. Notably, this is the first time to *ab initio* determine a nanocrystal HKUST-1 structure. During the structure determination, the high R_{int} and R_1 values are mainly caused by the dynamic effects of electrons. In the refinement, the structure factor was calculated in a kinematic method, while the electron diffraction data is dynamic. The details of data collection and refinement are summarized in Table S2. To validate the structures, we further simulated PXRD patterns of CUCAM-2 and HKUST-1, which match well with the experimental PXRD pattern (Figure S9). Because of the disorder and low occupancy, guest molecules in HKUST-1 were not determined. Thus, this causes the intensity difference of the first peak between the simulated HKUST-1 pattern and the experimental pattern.

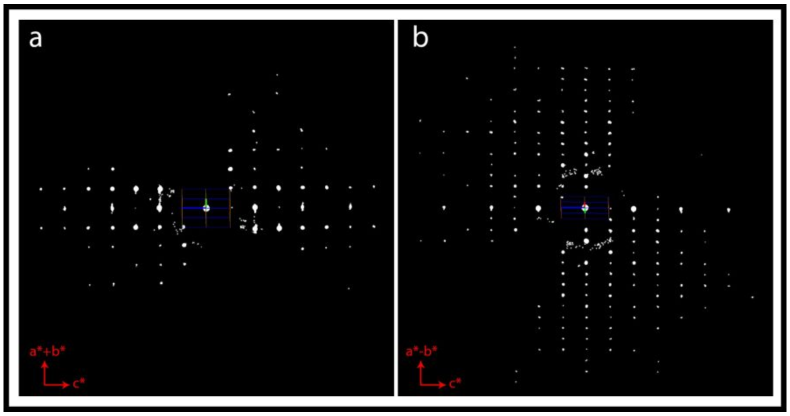

Figure S7. 2D slice cuts from the 3D reconstructed reciprocal lattice of CUCAM-2 (i) showing (a) hhl, and (b) h-hl planes.

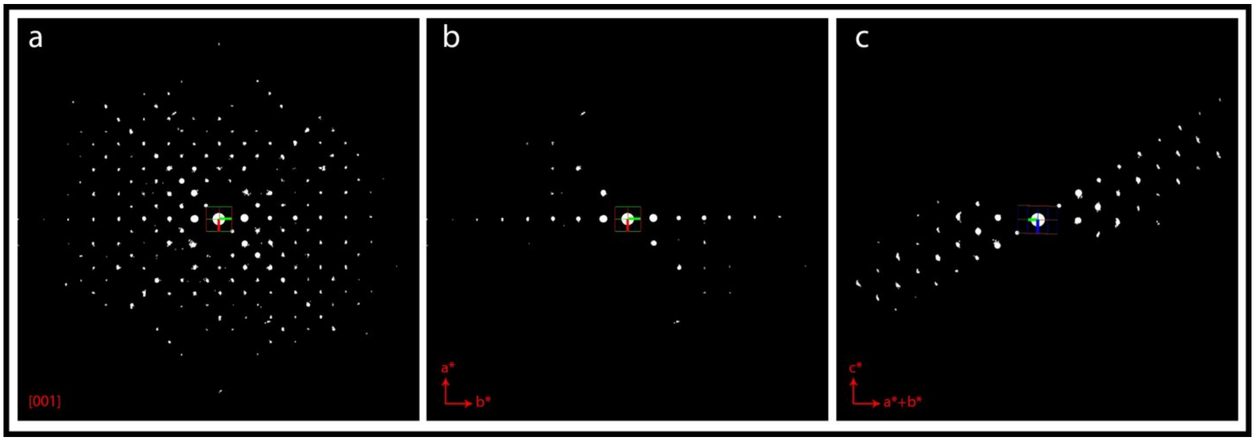

Figure S8. (a) The 3D reconstructed reciprocal lattice of CUCAM-2(i), viewing along c* axis.. 2D slice cuts from the 3D reconstructed reciprocal lattice of HKUST-1 nanocrystal showing (b) 0kl, and (c) hhl planes.

Table S2. Experimental parameters for cRED data collection and crystallographic data for CUCAM-2 phase 1 ((CUCAM—2)(i)) and phase 2 (HKUST-1) (λ = 0.0251 Å)

	**Phase 1 (CUCAM-2(i))**	**Phase 2 (HKUST-1)**
Chemical formula	$C_{13}CuN_2O_6$	$C_{12}Cu_2O_8$
Formula weight	339.71	399.20
Tilt range (°)	-52.0° to 60.5°	-4.4° to 40.7°
Tilt rate (°/s)	0.45	0.45
Exposure time/frame (s)	0.5	0.5
Total number of frames	437	148
Data collection time (min)	4.1	1.7
Resolution (Å)	0.70	1.30
Completeness	0.996	0.958
No. unique reflections	1871	274
No. observed reflections ($I > 2$ sigma(I))	1025	179
Crystal system	Hexagonal	Cubic
Space group	$P6_3/m$	Fm-3m
a/Å	16.354(2)	27.110(3)
c/Å	6.933(1)	= a
Z	6	24
Temperature/K	293(2)	293(2)
R_1 ($I > 2$ sigma(I))	0.217	0.173
R_1 (all reflections)	0.268	0.202
Goof	1.479	1.558

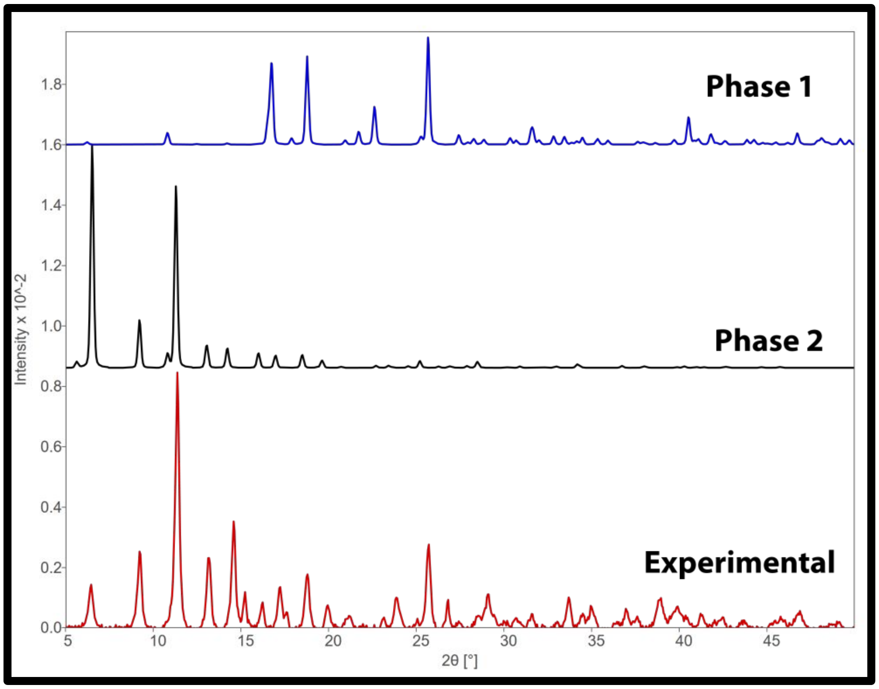

Figure S9. Experimental and simulated PXRD patterns (λ = 1.5418 Å) of Phase 1(CUCAM-2(i))and Phase 2 (HKUST-1) showing their coexistence in the green compound.

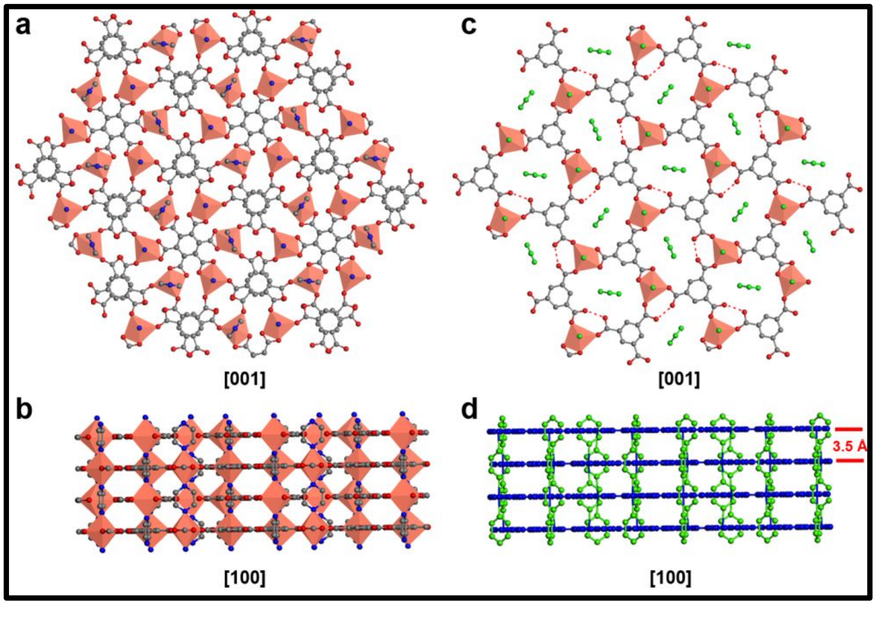

Figure S10. Structural model of CUCAM-2 viewing along (a) [001], and (b) [100] directions. (c) A single layer of CUCAM-2 shows coordination between Cu(II) and BTC, as well as hydrogen bonding between BTC. (d) Structural model of CUCAM-2 showing the 3D framework is composed by layers (blue) and pillars (green). Orange octahedral: Cu(II); grey spheres: C; red spheres: O; blue spheres: N.

5. Low-Pressure Gas Adsorption Measurements

Table S3 : Surface area and pore volume of CUCAM-2 after exchanging with F^-, Br^- & I^-

	Exchanged with		
	F-	Cl-	Br-
BET (m^2/g)	16	854	1540
Pore Volume (cc/g)	0.03	0.4	0.7

6. Thermogravimetric analysis

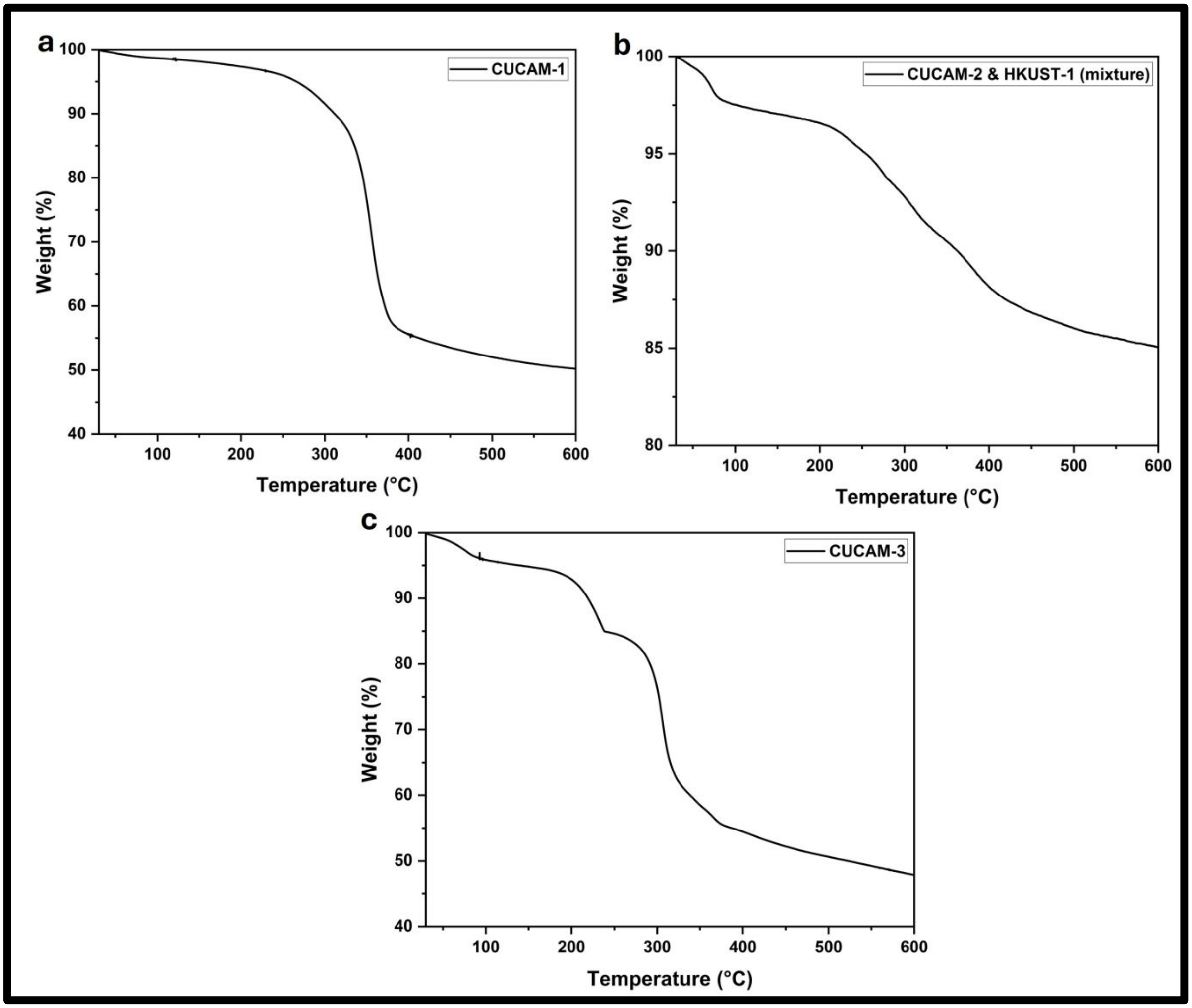

Figure S11. Thermogravimetric analysis of (a) CUCAM-1, (b) CUCAM-2 & HKUST-1 mixture, and (c) CUCAM-3

7. Anion Exchange-Assisted Recrystallization Transformation & Selective Iodide Detection

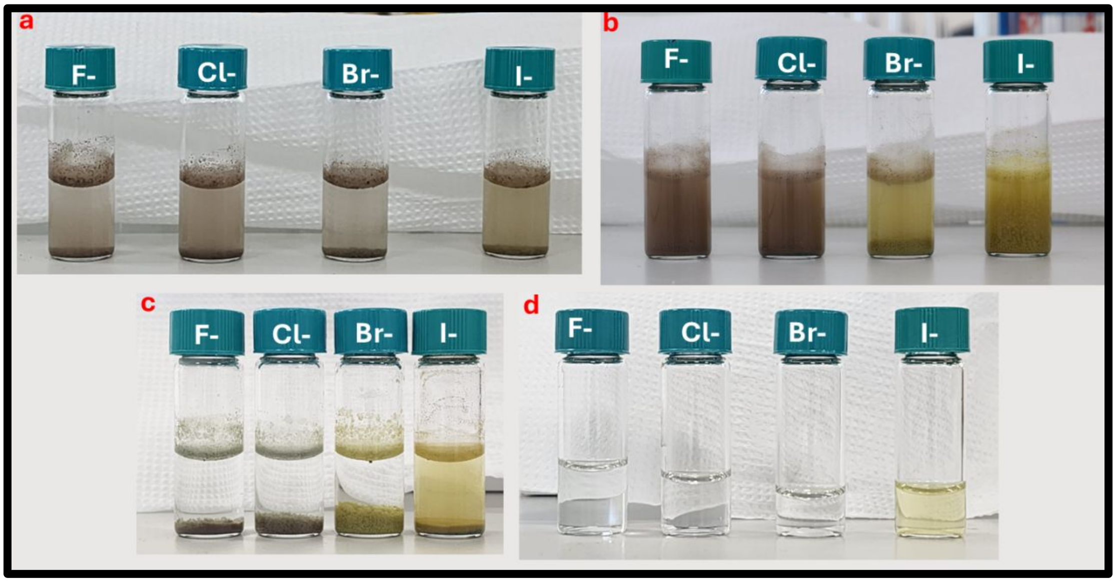

Figure S12. Color change of anion solution and **CUCAM-1** (a) immediately after the addition of CUCAM-1, (b) after 5 minutes, (c) after 15 hours, and. (d) the extracted anion solutions of each.

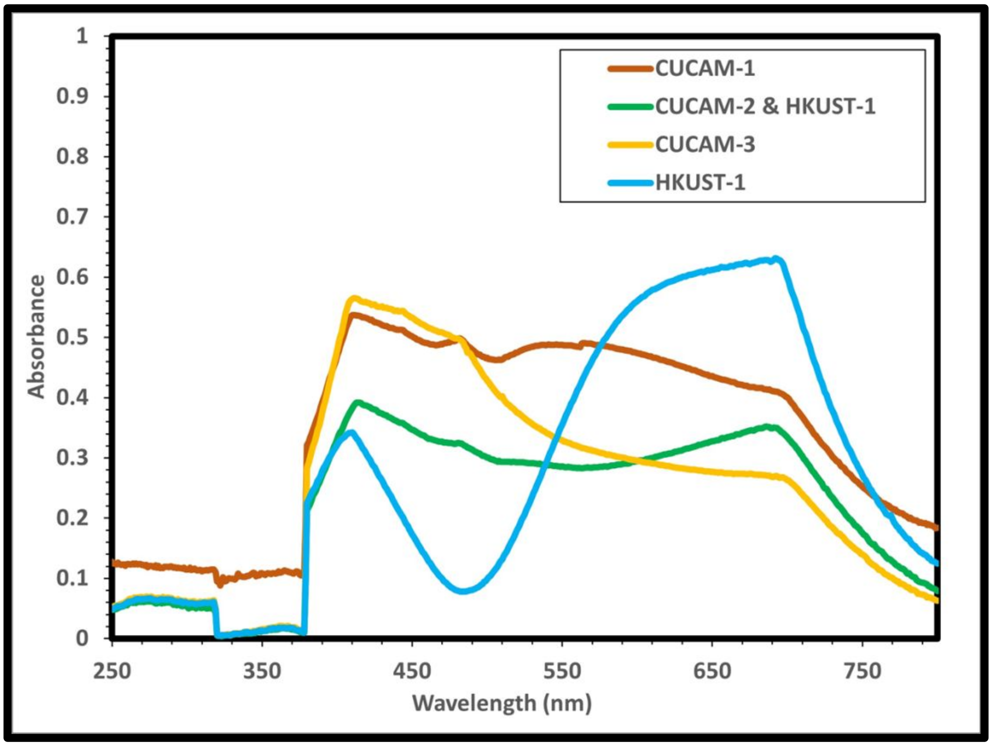

Figure S13. Solid state UV-Vis spectra of CUCAM-1, CUCAM-2 & HKUST-1 mixture (obtained by converting CUCAM-1), CUCAM-3 (by KI treatment), and HKUST-1 respectively.

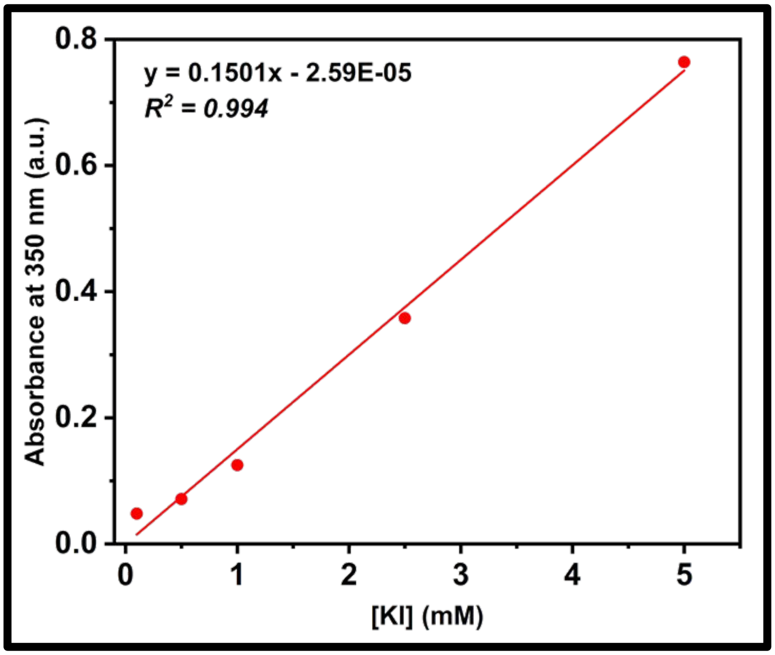

Figure S14. Absorbance (at 350 nm) vs. KI concentration calibration curve used for limit of detection determination of CUCAM-1 for iodide detection.

Table S4. LOD values of CUCAM-1 in comparison other reported colorimetric iodide sending MOFs.

**Sensor/detector**	**LOD (μM)**	**Reference**
CUCAM-1	50.2	This work
Co-bpe MOF	0.27	[3]
Cu-MOF	30.1	[4]

8. Reference

1 J. VandeVondele, M. Krack, F. Mohamed, M. Parrinello, T. Chassaing and J. Hutter, *Comput Phys Commun*, 2005, 167, 103–128.

2 J. P. Perdew, K. Burke and M. Ernzerhof, *Phys Rev Lett*, 1996, 77, 3865–3868.

3 D. Rani, K. K. Bhasin and M. Singh, *Dalton Transactions*, 2021, 50, 13430–13437.

4 N. Kaeosamut, Y. Chimupala, P. Yanu, S. Wannapaiboon, N. Sammawipawekul, S. Tonkaew, J. Jakmunee and S. Yimklan, *Inorg Chem*, 2022, 61, 19612–19623.
